# Complex Management of Basal Cell Carcinoma in a Frail Patient

**DOI:** 10.7759/cureus.53518

**Published:** 2024-02-03

**Authors:** Kawaiola Cael Aoki, Brad P Glick, Simona Bartos

**Affiliations:** 1 Dr. Kiran C. Patel College of Osteopathic Medicine, Nova Southeastern University, Fort Lauderdale, USA; 2 Dermatology, Larkin Community Hospital Palm Springs Campus, Margate, USA; 3 Dermatology, Imperial Dermatology, Hollywood, USA

**Keywords:** mohs surgery, dermato-surgery, dermato-oncology, watchful waiting, basal cell carcinoma (bcc)

## Abstract

Basal cell carcinoma (BCC) is one of the most common cancers diagnosed in older patients and has low mortality. Surgical versus medical management is considered in patients with multiple comorbidities and limited life expectancy (LLE), where the risk-to-benefit ratio must be carefully assessed. Watchful waiting (WW) is a viable option for some patients with severe LLE when follow-up care can be provided vigilantly and frequently. Special consideration should be given to morbidity factors such as tumor growth, bleeding, pain, and social withdrawal that negatively affect the quality of life. We present the case of a 75-year-old male with a past medical history of multiple system atrophy, who presented with a BCC on the ear and face. We discuss the management of this patient and factors that may have led to the inappropriate use of WW.

## Introduction

Skin cancer is the most common cancer diagnosed; over 50% of all skin cancers are diagnosed in patients over 65 [1]. Several factors contribute to the increasing prevalence of non-melanoma skin cancer (NMSC) in older adults - the overall increase in NMSC rates, the higher incidence of NMSC with age, and the aging global population [2]. By 2030, the overall incidence of NMSC is estimated to increase to 70% of the population [1]. Basal cell carcinoma (BCC) comprises approximately 80% of diagnosed skin cancer, is characterized by slow growth with low-malignant potential, and is often initially asymptomatic [1]. BCCs rarely metastasize (0.0029-0.55%) and may cause problems if left unattended for an extended period. Accordingly, the risk of treating BCCs may outweigh the benefits in adults with limited life expectancy (LLE) [3].

Although BCCs are generally indolent and rarely affect the quality of life (QoL), most are treated [4,5]. Up to 25% of patients report complications related to BCC treatment, especially in older patients with low QoL [6,7]. Watchful waiting (WW) entails monitoring the natural course of a disease without administering any active treatments, and these patients should be evaluated every three to six months. In the short term, WW circumvents the treatment burden and associated risks. However, WW may permit further tumor growth, resulting in severe tumor-related complaints and more complicated and invasive treatments [8]. Management options should be reconsidered in a WW approach at each follow-up visit, including re-evaluating if the medical justification for WW is still valid or if the risk-to-benefit ratio has altered.

## Case presentation

**Figure 1:**
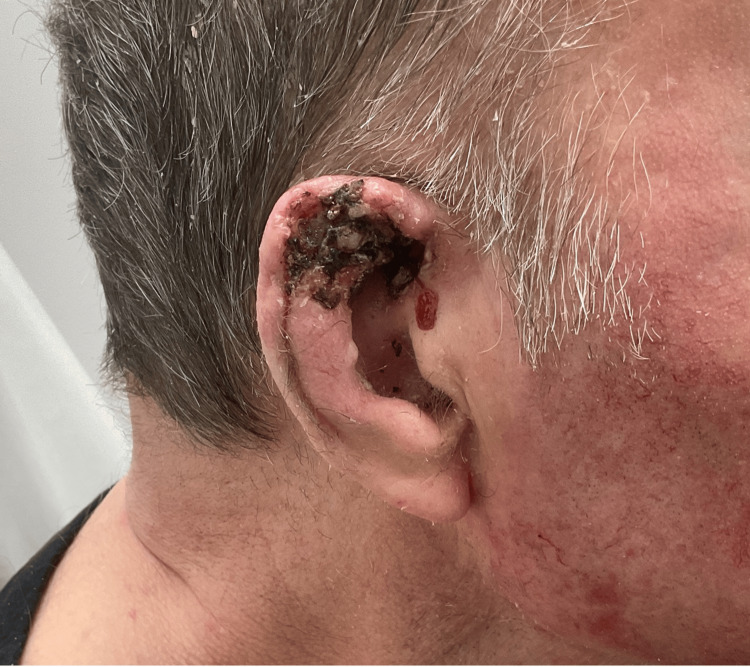
Initial presentation of basal cell carcinoma of the right ear

A 74-year-old Caucasian male with a past medical history of multiple system atrophy and BCC presented with an untreated BCC on the right ear. It had been biopsied three years prior by another dermatologist. Due to the COVID-19 pandemic and his comorbid conditions, WW was recommended. In the years that passed, the tumor grew, became tender, ulcerated, and started intermittently bleeding. On physical exam, there was a 4 by 3 cm (12 cm^2^) irregular lesion of the right ear involving most of the helix, crura, and scaphoid fossa and extending to the pre-auricular face (Figure 1). The patient and his wife voiced concerns over losing a large portion of the ear since he depended on oxygen via nasal cannula and needed to wear a mask during the pandemic.

A consultation with a radiation specialist was obtained. Superficial radiation therapy (SRT) is indicated for BCCs in poor surgical candidates, in cosmetically sensitive areas, in cases that would cause significantly impaired function and poor cosmesis, or in locally advanced disease extending into the cranial cavity. After discussion, it was deemed that the patient did not qualify for SRT as the treatment goals were unrealistic regarding the outcome of cosmesis of the ear (more fractions needed) and convenience (fewer fractions) due to his limited transportability.

**Figure 2:**
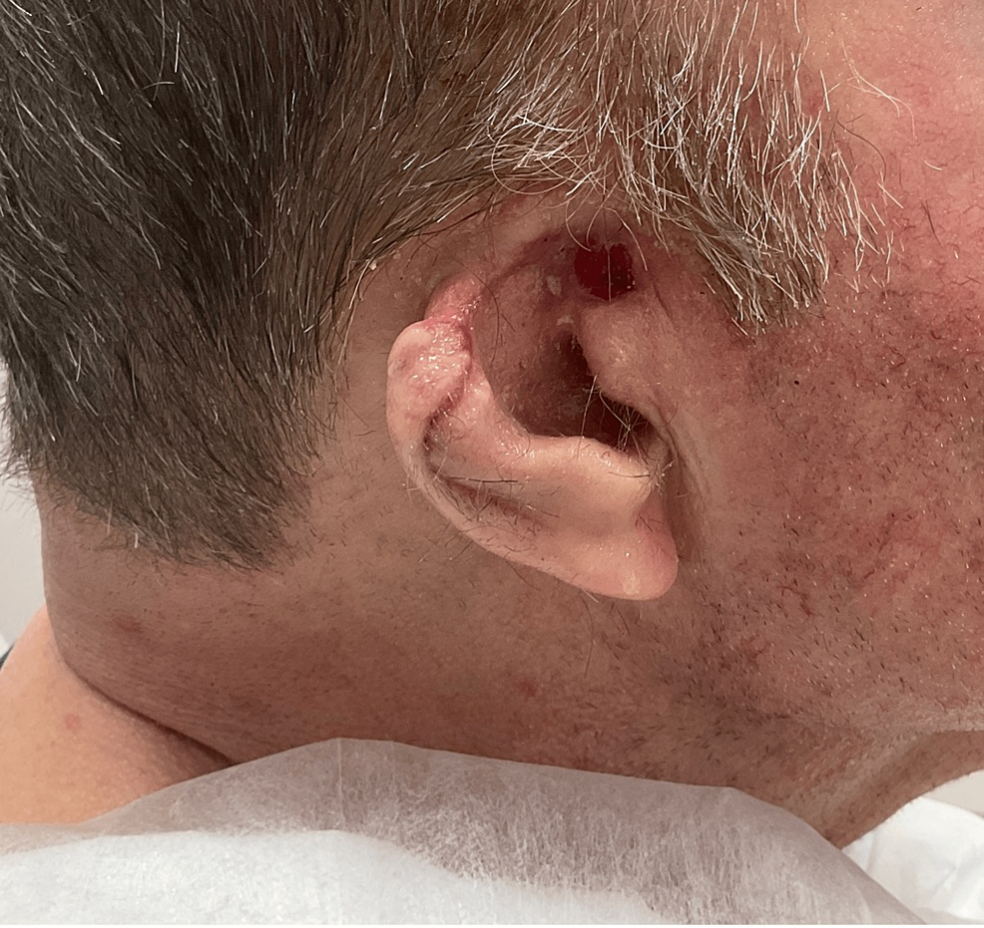
Healing defect at two weeks post-Mohs

Mohs micrographic surgery was recommended, and the patient underwent four Mohs stages. Due to discomfort, the patient did not wish to proceed and asked to stop any further surgical efforts. A plastic surgeon performed an advancement flap from the pina to the helix. The facial defect was further resected and approximated. Given the persistent tumor and the patient's desire to stop the surgery due to discomfort, the defect was closed by the consulting plastic surgeon. The remaining defect was covered with the skin substitute, PuraPly™. He received weekly dressing changes with PuraPly™ for the next four weeks and healed well without complications (Figure 2).

**Figure 3:**
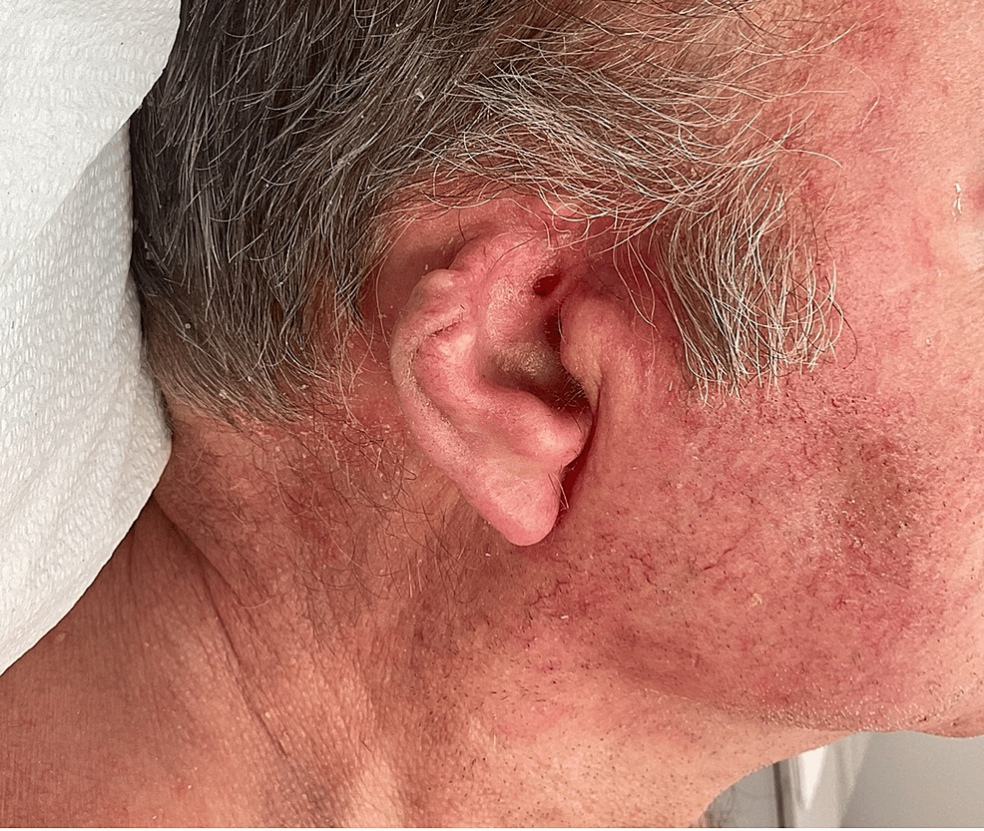
Recurrence of basal cell carcinoma at six-month follow-up

The patient was closely followed every three months. At six months, he had a biopsy-proven recurrence (Figure 3) and was treated with electrodesiccation and curettage (ED&C). The patient was offered systemic vismodegib but declined due to the high incidence of side effects reported with this medication. He was referred to radiation oncology, who is currently treating the patient.

## Discussion

The COVID-19 pandemic has created a unique challenge in managing this patient and has generally changed how care is delivered universally. Attributed to it is the increased use and permanence of telehealth, which poses a challenge to traditional, in-person care [9,10]. While most dermatological conditions can be appropriately treated via telemedicine, skin cancers deserve an in-person evaluation. Additionally, a significant consideration, in this case, included other practical barriers, such as time and availability of transportation. Thus, the frequency of follow-ups should align with what is manageable and feasible for the individual patient, such that they receive adequate care, but visits are not more onerous than the treatment burden attributed to the active treatment [8].

Despite BCC's low mortality and indolent growth pattern, there are no current guidelines or recommendations for WW or active surveillance as an evidence-based option for patients [11]. One study found that most (60.9%) of these BCCs were asymptomatic at initial presentation [8]. The most common symptoms included bleeding (36.5%), itch (25.0%), and crustae (23.1%). Eventually, 8% of BCCs developed symptoms during a median (interquartile range; IQR) follow-up time of 18 (9-15) months, but not all patients were bothered by the symptoms. This study found that 46.8% (of 280 BCCs) grew, approximately 4.46 mm per year for infiltrative/micronodular and 1.06 mm per year for other BCC subtypes. Other studies found similar results: 49% (of 39 BCCs) experienced an increase in tumor size after being monitored for an average of 15.8 months [11], and about half of the tumors, 47% (of 124 BCCs), increased in size with micronodular and infiltrative subtypes at higher risk of growth [12].

After an initial WW approach, 38.2% of BCCs (n=107) in 63% of patients (n=54) were eventually treated, and the median (IQR) time till treatment was 7 (5-11) months. More invasive treatment than estimated at initial presentation, such as reconstruction (vs. primary closure) or Mohs (vs. conventional excision), was required in 2.8% of BCCs. More concerning was that this study found a discrepancy between the initial biopsy histological subtype and the histological subtype identified after excision. Of these eight tumors initially classified as low-risk subtypes, six were found to be mixed nodulo-infiltrative, and two were squamous cell carcinoma [8]. Overall, the literature indicates that patients with advanced age and comorbidities endure surgical treatment well, and an LLE had no impact on treatment choices for patients with NMSC, including those undergoing Mohs surgery [4,6,13].

A holistic approach would lead to different BCC management choices in complex cases such as these. Specific attention should be paid to patient-related factors and treatment goals that coincide with the values and preferences of each patient, particularly when life expectancy and time to benefit are roughly equivalent. Patient satisfaction is not necessarily analogous to treatment burden, as patients can express satisfaction with their care while still experiencing a significant treatment burden that affects their daily activities and social resources [13]. In one study, 65% of patients mentioned treatment goals and preferences other than curative treatment, such as symptom relief, least burdensome treatment, cosmetic goals, or no treatment [14]. Thus, considering all management options, clinicians may opt for less aggressive treatment options for frail older adults whose individual treatment goals may not be curative versus curative approaches for those individuals who will benefit from them. To this end, clinical decision-making tools, such as predictive instruments, may assist in identifying patients needing multidisciplinary approaches, extensive counseling, or complex management [14].

Frailty is defined as "vulnerability and physical deterioration, which leads to a disproportionally decreased ability to cope with stressors" [15]. Frailty-related characteristics, including comorbidity, cognition, and functional status, should be considered in medical decision-making [16]. However, current clinical guidelines for treating BCCs are based primarily on tumor location, size, and histologic type. A patient-centered treatment approach in older adults that incorporates life expectancy, frailty, and comorbidities is needed [17,18]. Predictive instruments to determine the degree of frailty and a patient's overall prognosis can aid in making management decisions; however, these tools currently lack validation for patients with NMSC [19]. Nonetheless, patients with LLE may benefit from an extended time for evaluating patient-related factors, such as implementing the Geriatric-8 (G8) frailty screening tool [20,21].

Frailty-related patient characteristics are significant predictors of higher treatment burden and overall mortality. In multivariable regression, the predictors of higher treatment burden were dependence on Instrumental Activities of Daily Living (iADL), female sex, larger tumor diameter, and polypharmacy. Additionally, significant predictors of complications included tumor diameter (OR=1.07, 95% CI: 1.03-1.11, p=0.001) and wound closure technique. Compared to primary closure, there was an increased OR of complications in closure through secondary intention (OR=2.69, 95% CI: 1.19-6.10, p=0.017) and reconstruction (OR=4.98, 95% CI: 2.49-9.98, p<0.001). An increasing number of comorbidities and dependence on iADL were also significantly linked to an overall rise in short-term mortality not related to BCC [13]. However, estimating life expectancy remains challenging, as another study indicated that comorbidity was less accurate at estimating five-year survival [4].

One method of addressing the unique needs of these patients is the use of shared decision-making, which involves four components: 1) the participation of both the physician and patient; 2) the sharing of information between the two parties; 3) both parties collaboratively identifying the preferred treatment option; and 4) reaching a treatment agreement [22]. Patient decision aids (PDAs) can aid this process by using simple numbers and visuals that are easy for patients to understand [23]. The parties can communicate risk and uncertainty, disclose aspects of the treatment not necessarily found in the medical literature (such as the length of the procedure), and allow for values clarification, a process that enables patients to incorporate their personal values and preferences into the decision-making process [24]. Patients preferred sample narratives to clarify their preferences when making decisions that involve several treatment options with varying trade-offs [25]. Lastly, physicians should be cognizant of information framing, as patients place significant reliance on how information is presented and formulated [26].

Because BCC's natural disease progression is unpredictable, estimating the risk-to-benefit ratio must be tailored to the specific patient and family, considering comorbidities, life expectancy, surgical risk, and personal preferences. Close monitoring is crucial, and if these slow-growing tumors become burdensome, surgical resection should be implemented even when the goal is only palliation. More data is needed to guide clinical decision-making when WW may be more appropriate than treating BCCs. Prior research has shown that LLE, frailty, and patient preference were important factors for choosing WW; integrating these factors into a risk to benefit that guideline remains limited [16,27,28]. Currently, there is little data to guide clinical decision-making in situations where WW may be more appropriate for management [16,29].

## Conclusions

Treatment for BCC includes surgery, either excisional or Mohs, ED&C, radiation, or chemotherapy. WW can be implemented in patients with significant comorbidities, LLE, or poor surgical candidates (due to anesthesia risk or prolonged immobilization). If WW is chosen, these patients must be re-examined frequently. When treating patients with cutaneous carcinomas, clinicians need to consider multiple patient and disease progression factors, including permanent and functional changes resulting from surgery, in the current case, a partial ear amputation. A detailed discussion with patients regarding the extent of surgery and possible disfigurement is paramount for informed consent. Balancing risks and benefits for treatment should be done in a timely fashion to avoid disease progression that may significantly decrease the quality of life in a patient, such as described above.

The authors believe advanced and rapidly growing tumors should be treated with surgery for curative, salvage, or palliative intent. Early surgical intervention that would have extirpated the cancer may have prevented significant pain and suffering to this patient and his family. Additionally, it would save considerable healthcare dollars for the complex surgery and reconstruction necessary to resect a much larger tumor. The art of medical practice shines when we prescribe a tailored plan to every unique patient and avoid classifying patients into specific treatment categories based on age, medical comorbidities, and LLE. A timely surgery may just be what the "doctor ordered."
